# Prediction of difficult laryngoscopy / difficult intubation cases using upper airway ultrasound measurements in emergency department: a prospective observational study

**DOI:** 10.1186/s12873-023-00852-4

**Published:** 2023-07-25

**Authors:** Mehran Sotoodehnia, Maryam Khodayar, Alireza Jalali, Mehdi Momeni, Arash Safaie, Atefeh Abdollahi

**Affiliations:** 1 Department of Emergency Medicine, Sina Hospital, Tehran University of Medical Sciences, Tehran, Iran; 2 Imam Hossein Center for Education Research and Treatment, Shahroud University of Medical Sciences, Shahroud, Iran; 3 Department of Emergency Medicine, Shariati Hospital, Tehran University of Medical Sciences, Tehran, Iran; 4 Department of Emergency Medicine, Sina Hospital, Terhran, Iran

**Keywords:** Airway Management, Intubation, Intratracheal, Laryngoscopy, Ultrasonography

## Abstract

**Introduction:**

Difficult laryngoscopy and intubation are serious problems among critically ill patients in emergency department (ED) so utility of a rapid, accurate and noninvasive method for predicting of these patients are necessary. Ultrasonography has been recently used in this regard and this study was conducted to investigate the correlation of some introduced upper airway ultrasound parameters with difficult laryngoscopy / difficult intubation in patients referred to the ED.

**Method:**

In this prospective observational study all patients ≥ 18-year-old who had an indication for rapid sequence intubation (RSI) were included. Ultrasound parameters including Hyoid Bone Visibility (HBV), Distance from Skin to Hyoid Bone (DSHB), Distance from Skin to Vocal Cords (DSVC), Distance from Skin to Thyroid Isthmus (DSTI), and Distance between Arytenoids Cartilages (DBAC) were measured in all cases. The patients underwent RSI and thereafter the patients’ baseline characteristics, Cormack-Lehane grade, number of attempted laryngoscopy were recorded in a pre-prepared check list and compared with measured ultrasound parameters. The “difficult laryngoscopy” was defined as Cormack-Lehane classification grades III/IV; and need for more than 3 intubation attempts was considered as “difficult intubation”.

**Results:**

One hundred and twenty-three patients (52% male) were included of whom 10 patients (8.1%) were categorized as difficult laryngoscopy cases; and just 4 (3.3%) cases underwent more than 3 laryngoscopy attempts who considered as difficult intubation cases. The mean age of the patients in non-difficult and difficult intubation groups were 69.2 ± 15.16 and 68.77 ± 17.37 years, respectively (p > 0.05). There was no significant relationship between difficult laryngoscopy and HBV (p = 0.381) but has significant correlation with difficult intubation (p = 0.004). The DSHB had a significant correlation with difficult laryngoscopy (p = 0.002) but its correlation with difficult intubation was not significant (p = 0.629). The DSVC and DSTI had a significant relationship with both difficult laryngoscopy (p = 0.003 and p = 0.001), and difficult intubation (p = 0.025 and p = 0.001). The DBAC had not significant correlation neither with the difficult laryngoscopy (p = 0.142), nor with difficult intubation (p = 0.526).

**Conclusion:**

The findings showed that ultrasound parameters including soft tissue DSHB, DSVC and DSTI could be proper predictors of difficult laryngoscopy. Also, HBV, DSVC and DSTI may be proper predictors for difficult intubation. But DBAC was not useful in this regard.

## Introduction

Tracheal intubation (TI) through direct laryngoscopy is often performed in patients to establish an airway to provide adequate ventilation and oxygenation, and/or to protect the airway from aspiration of oral and pharyngeal secretions [1, 2]. Difficult intubation occurs due to insufficient vision of the larynx during direct laryngoscopy and its incidence in elective cases in operating rooms is varying from 0.4 to 8.5% [3, 4]. But in emergency departments (ED), the incidence is higher and reach about 14.8% [5–7]. Some constraints such as full stomach and aspiration risk, unknown past medical and allergy history may make the actual number of difficult intubation in the ED higher than the number reported by anesthesiologists in operating room [8–11]. It seems that, to prevent complications due to repeated attempt for intubation (arrythmia, hypoxia, …), early detection of probable difficult laryngoscopy cases is of great importance in the ED. Therefore, various screening methods and scales have been defined in this regard [12, 13]. Cormack-Lehane classification, Wilson’s Criteria, Macocha score are among screening methods that is used to predict difficult airway and laryngoscopy cases; However, all have considerable limitations [3, 14, 15]. Therefore, the search for a simple, non-invasive technique that provides a more accurate assessment of the patient’s airway still continues. The ideal method is expected to be fast, accessible, simple and non-invasive [11, 16, 17]. Today, portable ultrasound devices are widely available in EDs and recently studies have focused on its capabilities in terms of airway management [18]. At present, airway ultrasonography is not yet used as a common method for airway assessment. Although several parameters of airway ultrasound have been mentioned in various studies as difficult airway prediction indicators, research is still ongoing to obtain easy and accurate measures [15, 19]. Therefore, this study performed to investigate the relationship between some upper airway ultrasound assessment parameters with difficult laryngoscopy / difficult intubation in patients referred to the ED and aim to use these parameters to assist physician to decide about difficult laryngoscopy/ difficult intubation and consider as predictors beside the traditional methods.

## Methods

### Study setting and population

This prospective observational study was conducted prospectively for a period of one year from May 2019 to May 2020 in the ED of educational medical centers (Shariati and Sina Hospitals) in Tehran, Iran.

All patients who were in age range of 18–80 years, who had referred to the EDs of mentioned hospitals, with an indication for performing rapid sequence intubation (RSI), were eligible. Patients with trauma to the neck, face or those required cervical collars or had indication of crash intubation, and also patients with clear airway obstruction were excluded. We calculated the required sample size based-on the comparison of the area under a ROC curve (AUC) of ultrasound parameters in predicting difficult laryngoscopy with a null hypothesis value. So, we to show that the assumed AUC of 0.75 for ultrasound parameters is significant from the null hypothesis value 0.5, and 10% of difficult laryngoscopy, 0.05 of Type I error – alpha and power with 90%, the required sample size was 123 patients.

### Definitions

The “difficult laryngoscopy” based on ASA Task Force was defined as Cormack-Lehane classification grades 3 and 4. Need for more than 3 intubation attempts by a trained provider or attempts at intubation that last longer than 10 min was considered as “difficult intubation”.

### Ultrasonography technique

Airway ultrasonography was performed using the Linear probe 6-13^MHZ^ and the ultrasound device SONOACE X8 SAMSUNG, by a post-graduation year-3 (PGY-3) emergency medicine resident who had been trained for 2 months in the airway ultrasound workshop to determine ultrasound parameters. Sonography was performed in cardiopulmonary resuscitation (CPR) room in the ED, during conducting pre-oxygenation phase of RSI and without interfering with it. The patient was in a supine position with the head extended from the neck and the neck was in an angled or curved position relative to the trunk. The airway was examined in the anterior neck in two views (i) Sagittal view on the longitudinal axis of the middle line and (ii) Transverse view in anterior of the neck. Five ultrasound parameters including Hyoid Bone Visibility (HBV), Distance from Skin to Hyoid Bone (DSHB), Distance from Skin to Vocal Cords (DSVC), Distance from Skin to Thyroid Isthmus (DSTI), and Distance between Arytenoids Cartilages (DBAC) were measured and recorded. Then, the patient was intubated by the in-charge physician. Macintosh blades were used for intubation in all patients. All cases of intubation were performed by a senior student of emergency medicine.

### Data collection

After performing the ultrasound and RSI, the patient’s information including age, sex, body mass index (BMI), neck circumference size at the superior border of the thyroid cartilage, Cormack-Lehane grade, and the number of performed laryngoscopies were recorded in a pre-prepared checklist.

### Statistical analysis

Central indicators (mean, median, etc.) and dispersion indicators (standard deviation (SD), confidence interval (CI), variance, etc.) were used to analyze descriptive data. Comparison analysis was performed using the independent t-test for continuous variables and chi-square or Fisher exact test for non-continuous variables. We used of Kolmogorov–Smirnov test and graphical approaches, like Q-Q plot for assess of normality assumption in continuous variables for using parametric or non-parametric test. Receiver operating characteristic curve (ROC) analyses were used to calculate the comparable threshold values of ultrasound parameters. SPSS-20 software was used to analyze the data. The level of statistical significance was p-value < 0.05 in all statistical analysis.

## Results

### Baseline findings

In this study, 123 patients, including 64 males (52%) and 59 females (48%), were evaluated, whose baseline variables are summarized in Table 1. Ten out of 123 patients (8.1%) had difficult laryngoscopy. Females accounted for 60% of patients with difficult laryngoscopy, and there was no significant correlation between gender or age and difficult laryngoscopy. It should be noted that 119 (96.7%) patients intubated with less than 3 laryngoscopy attempts, and just 4 (3.3%) underwent more than 3 laryngoscopies who considered as difficult intubation cases.

**Table 1:** Baseline variables of the two groups of difficult and non-difficult laryngoscopy cases

Variable	Difficult (n = 10)	Non-difficult (n = 113)	p
	Mean ± SD / number (%)		
**Age (year)**	69.2 ± 15.16	68.8 ± 17.4	0.4
**Sex**			0.4
Male	4 (40.0)	60 (53.1)	
Female	6 (60.0)	53 (46.9)	
**Neck circumflex (cm)**	43.73 ± 4.89	39.43 ± 4.52	0.005
**Body Mass Index**	32.7 ± 5.56	24.67 ± 4.95	0.001
< 18.5	0 (0.0)	6 (5.3)	
18.5–24.9	2 (20.0)	62 (54.9)	
25–29.9	5 (50.0)	40 (35.4)	
≥ 30	3 (30.0)	5 (4.4)	
**Cormack-Lehane classification**			< 0.001
Grade 1	0 (0.0)	69 (61.1)	
Grade 2	0 (0.0)	44 (38.9)	
Grade 3	7 (70.0)	0 (0.0)	
Grade 4	3 (30.0)	0 (0.0)	

### Difficult laryngoscopy

The relationship of the measured ultrasound parameters with difficult laryngoscopy is reported in Table 2. Based on the findings, DSHB, DSVC, and DSTI had a significant relationship with difficult laryngoscopy (p < 0.05). However, there was no significant relationship between DBAC and difficult laryngoscopy (p = 0.142). The hyoid bone was not visible in 13 patients (10.6%), that just 3 of them had Cormack-Lehane grade III/IV; and also hyoid bone was visible in 7 out of 10 difficult laryngoscopy cases. These findings indicate that there was no significant relationship between difficult laryngoscopy and HBV (p = 0.381).

**Table 2:** The relationship of the ultrasound parameters with difficult laryngoscopy

Variable	Difficult (n = 10)	Non-difficult (n = 113)	p
	Mean ± SD (mm)		
**DSHB**	11.04 ± 2.06	8.60 ± 1.93	0.002
**DSVC**	9.42 ± 1.66	7.58 ± 1.58	0.003
**DSTI**	11.55 ± 2.17	8.75 ± 2.22	0.001
**DBAC**	7.17 ± 1.92	6.38 ± 1.59	0.142

HBV: Hyoid Bone Visibility. DSHB: Distance from Skin to Hyoid Bone. DSVC: Distance from Skin to Vocal Cords. DSTI: Distance from Skin to Thyroid Isthmus. DBAC: Distance between Arytenoids Cartilages

**Fig. 1:**
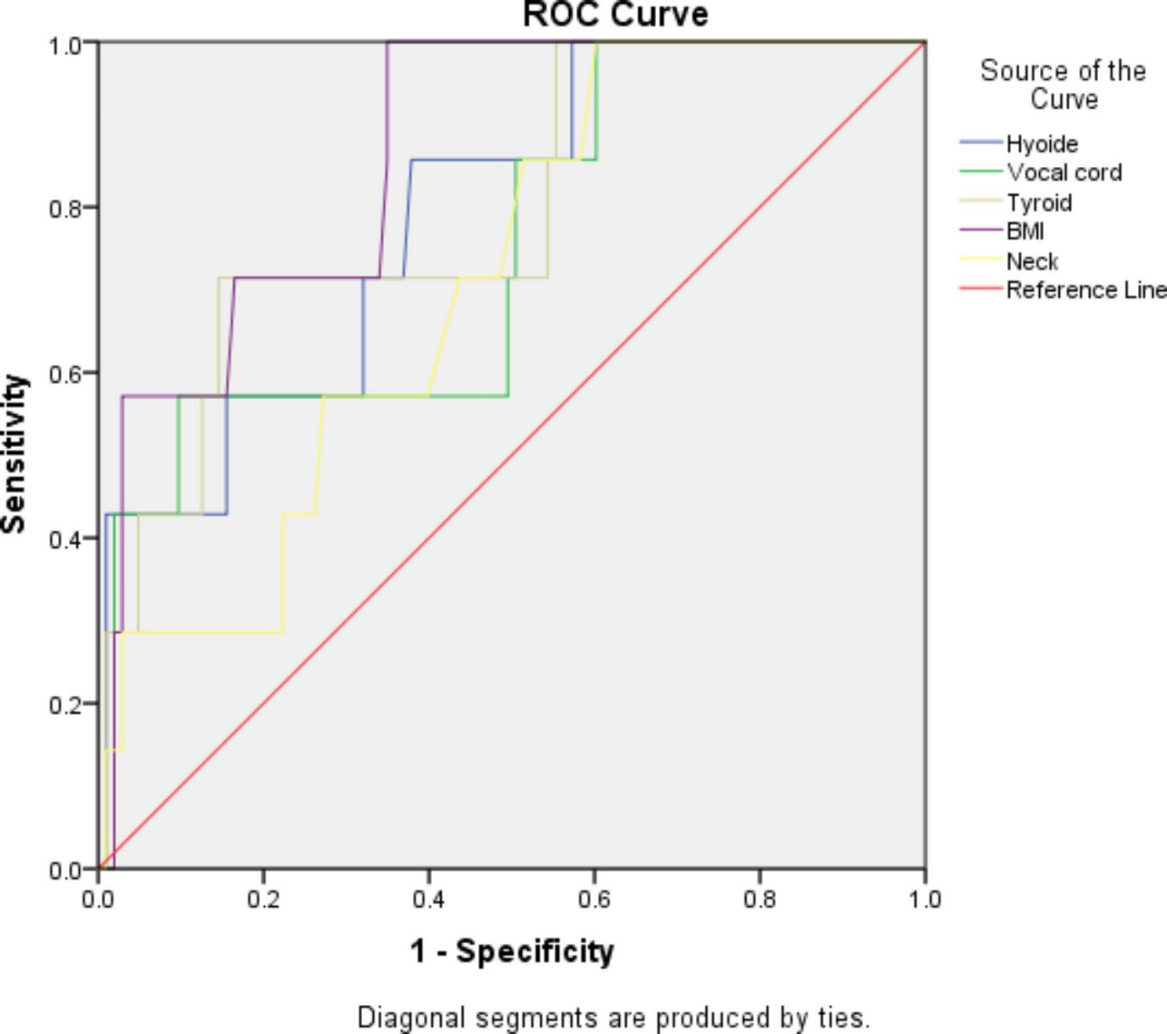
ROC graph of ultrasound parameters

The ROC curve was used to determine the best cut off point of indices (Fig. 1). AUC of all parameters was higher than 0.7, indicating that all of them were appropriate parameters in predicting difficult laryngoscopy. The sensitivity of DSHB, DSVC and DSTI parameters in estimating difficult laryngoscopy were 57%, 70%, and 80%, respectively, and their specificity in the diagnosis of difficult laryngoscopy were 84%, 84%, and 77%, respectively. The cut of point number for DSHB, DSVC, and DSTI was 10.33, 9.41 and 10.16 mm, respectively. The DSTI index had the highest sensitivity and DSHB and DSVC had the highest specificity in predicting difficult laryngoscopy (Table 3).

**Table 3:** Statistical characteristics of measured ultrasound parameters in predicting difficult laryngoscopy

Variable	Cut of point (mm)	Sensitivity	Specificity	Confidence interval Higher bound	Confidence interval Lower bound	AUC
**DSHB**	10.33	57%	84%	0.952	0.633	0.793
**DSVC**	9.41	70%	84%	0.928	0.623	0.776
**DSTI**	10.16	80%	77%	0.944	0.670	0.807

DSHB: Distance from Skin to Hyoid Bone. DSVC: Distance from Skin to Vocal Cords. DSTI: Distance from Skin to Thyroid Isthmus

The sensitivity and specificity of HBV in the upper airway ultrasound was, 91.15% (CI = 95.84–67.33) and 30% (CI = 65.25–6.67), respectively. The positive and negative predictive values of HBV were 93.64% (CI = 95.68–90.71) and 23.08% (CI = 47.81–8.94), respectively (Table 4).

**Table 4:** Statistical characteristics of hyoid bone visibility via ultrasound in predicting difficult laryngoscopy

Characteristic	Results	Higher bound confidence interval	Lower bound confidence interval
**Sensitivity**	91.15%	95.67	84.33
**Specificity**	30%	65.25	6.67
**Positive likelihood ratio**	1.30	1.96	0.86
**Negative likelihood ratio**	0.29	0.90	0.10
**Positive predictive value**	93.64%	95.68	90.71
**Negative predictive value**	23.08%	47.81	8.94

### Difficult intubation

Difficult intubation rate in this study was 8.1%. There was no failed intubation among samples, and patients with difficult intubation were eventually intubated by another method, such as using a bougie. Table 5 shows the statistical relationship between the 5 ultrasound parameters and difficult intubation. Among patients who were not difficult intubation cases, the hyoid bone was not visible in 10 (8.4%) patients. In those with difficult intubation, the hyoid bone was not visible in 3 (75%) patients, indicating a significant relationship between HBV and difficult intubation (p = 0.004). However, the difficult intubation had no significant relationship with DSHB and DBAC. But difficult intubation had a significant relationship with DSVC and DSTI (p < 0.05).

**Table 5:** Statistical characteristics of measured ultrasound parameters in predicting difficult intubation

Variable	Number of laryngoscopy attempts		p
	< 3	≥ 3	
	Mean ± SD / number (%)		
**HBV**			0.004
Yes	109 (91.6%)	1 (25%)	
No	10 (8.4%)	3 (75%)	
**DSHB (mm)**	8.75 ± 2.02	9.74 ± 1.05	0.629
**DSVC (mm)**	7.66 ± 1.87	9.81 ± 1.54	0.025
**DSTI (mm)**	8.85 ± 2.24	12.78 ± 1.85	0.001
**DBAC (mm)**	6.43 ± 1.64	6.96 ± 1.48	0.526

HBV: Hyoid Bone Visibility. DSHB: Distance from Skin to Hyoid Bone. DSVC: Distance from Skin to Vocal Cords. DSTI: Distance from Skin to Thyroid Isthmus. DBAC: Distance between Arytenoids Cartilages

## Discussion

### Difficult laryngoscopy

In this study, ultrasound was able to predict difficult laryngoscopy and difficult intubation cases in a significant way, so that it showed that 3 indices of DSHB, DSVC, and DSTI were significantly valuable in predicting the presence of difficult laryngoscopy. Among them, DSTI had the highest sensitivity, and DSHB and DSVC had the highest specificity in terms of predicting difficult laryngoscopy.

### Difficult intubation

These findings are consistent with Adhikari et al. [20] and Wojtczak [21] results. In a study by Adhikari et al. [20], DSHB in easy and difficult intubation cases was 1.37 vs. 1.69 cm and distance from skin to anterior thyrohyoid membrane was 2.37 vs. 3.47 cm, significantly indicating the relationship between difficult intubation and the distance from skin to the hyoid bone and anterior thyrohyoid membrane. In this study, the hyoid bone was visible in 70% of patients with difficult intubation. In a study by Hui et al. [22], 11% of patients had difficult intubation. The hyoid bone was visible in 96.6% of patients with easy intubation, and not visible in 72.7% of patients with difficult intubation. This inconsistency may be due to differences in ultrasound techniques and device quality, BMI, or racial differences in patients. Our patients were of East Asian descent, while most studies have been conducted in European and American races.

In our study, DSHB and DSVC had the highest specificity (84%) which is consistent with results from Parameswar et al. [14], Adhikari et al. [20] and Kumatsu et al. [23], examining airways of 64 patients with overweight and BMI > 35 using ultrasound who had difficult intubation found that DSVC (20.3 ± 4 mm) in these patients was shorter than that of patients with easy intubation (22.3 ± 3.8 mm) [23].It appears that according to previous studies and comparing their results with the current study, DSHB and DSVC are reliable factors for predicting difficult intubation. According to Hui CM, HBV was recognized as a strong predictive factor in determining difficult intubation with 73% sensitivity and 97% specificity [22]. According to similar results in the present study, HBV can be a predictive factor for difficult intubation. We recommend that anterior neck soft tissue (distance from the skin) should be measure at two levels to predict difficult intubation using ultrasound: the distance from skin to the hyoid bone and the vocal cords. HBV shall also be examined; and it is also recommended to use ultrasound as a difficult intubation screening tool along with clinical screening methods.

In a 2015 systematic study, Bajracharya et al. [24] examined the accuracy of airway assessment by ultrasound during anesthesia. The study found that ultrasound, like computed tomography (CT) scans and magnetic resonance imaging (MRI) could show high-resolution images of the anatomical structures of the upper airway. Various ultrasound parameters, including distance from skin to the hyoid bone, epiglottis and vocal cords; HBV in sublingual ultrasound, and hyomental distance were also identified as independent predictors of difficult laryngoscopy in obese and non-obese patients, also this indicator was found as the most consistent predictor in a systematic review conducted by Gomes et al. [25]. CT scan and MRI can also measure the anterior neck soft tissue thickness, but are expensive and not available in many operating rooms, while portable ultrasound is a cheap, affordable, and fast way to assess the airway.

In this study DBAC had no significant correlations with difficult intubation, neither with the number of laryngoscopies of greater than or equal to three times, or the Cormack-Lehane grade. There is no similar study to compare the results. The low sample size in this study can also affect the results, so it is recommended to conduct further studies with a larger sample size to investigate the relationship between DBAC and difficult intubation.

In this study, ultrasound parameters were performed at three levels on the anterior neck and showed that ultrasound can be used to assess the airway and it is possible to measure 5 factors before intubation by ultrasound within 3–5 min.

### Limitations

This study also had some limitations. Intubation is a complex procedure and many factors such as skill and experience of the physician, presence of secretions and blood in the airway, and presence of airway abnormalities interfere with its successful performance. Moreover, patient agitation and restlessness, lack of cooperation of patients in emergency conditions, overcrowding and the presence of multiple critically ill patients in the emergency room at the same time, and not having enough time to perform an ultrasound on the bed of hemodynamically unstable patients can interfere with having an ultrasound before intubation. Ultrasound is a tool that depends on the operator. The experience and skill of the physician who uses it is very effective in the results. Conducting this study with a larger sample size can more accurately show the relationship of ultrasound parameters of the upper airway with the degree of difficulty of intubation. The intubating person’s prior knowledge of factors influencing difficult intubation before performing an ultrasound may be effective in measuring the parameters of the ultrasound.

## Conclusion

According to the results of this study, ultrasound parameters including soft tissue DSHB, DSVC and DSTI could be considered as proper predictors of difficult laryngoscopy. Also, HBV, DSVC and DSTI may be considered as proper predictors for difficult intubation. While DBAC was not useful neither for predicting difficult laryngoscopy, nor difficult intubation, DSVC and DSTI were useful in predicting both difficult laryngoscopy and difficult intubation cases. According to our study, clinicians can deploy these useful indicators as predictors of difficult intubation / difficult laryngoscopy to decide at the bedside before acting on intubation.

## Data Availability

All data would be available via contacting the corresponding author.

## Abbreviations

AUC: The area under a ROC curve
BMI: Body mass index
CI: Confidence interval
CPR: Cardiopulmonary resuscitation
CT: Computed tomography
DBAC: Distance between arytenoids cartilages
DSHB: Distance from skin to hyoid bone
DSTI: Distance from skin to thyroid isthmus
DSVC: Distance from skin to vocal cords
ED: Emergency department
HBV: Hyoid bone visibility
MRI: Magnetic resonance imaging
PGY-3: Post-graduation year-3
ROC: Receiver operating characteristic curve
RSI: Rapid sequence intubation
SD: Standard deviation
TI: Tracheal intubation
